# Shedding Light on the Possible Link between ADAMTS13 and Vaccine—Induced Thrombotic Thrombocytopenia

**DOI:** 10.3390/cells10102785

**Published:** 2021-10-18

**Authors:** Anna Szóstek-Mioduchowska, Paweł Kordowitzki

**Affiliations:** 1 Department for Immunology and Pathology, Institute of Animal Reproduction and Food Research of Polish Academy of Sciences, Tuwima Street 10, 10-243 Olsztyn, Poland; a.szostek-mioduchowska@pan.olsztyn.pl; 2 Faculty of Biology and Veterinary Medicine, Nicolaus Copernicus University, Gagarina Street 1, 87-100 Torun, Poland

**Keywords:** SARS-CoV-2, COVID-19, vaccine, thrombosis, ADAMTS13, platelet factor 4, thrombocytopenia, von Willebrand factor

## Abstract

Several recent reports have highlighted the onset of vaccine-induced thrombotic thrombocytopaenia (VITT) in some recipients (approximately 1 case out of 100k exposures) of the ChAdOx1 nCoV-19 vaccine (AstraZeneca). Although the underlying events leading to this blood-clotting phenomenon has yet to be elucidated, several critical observations present a compelling potential mechanism. Thrombus formation requires the von Willebrand (VWF) protein to be in ultra-large multimeric state. The conservation of this state is controlled by the ADAMTS13 enzyme, whose proteolytic activity reduces the size of VWF multimers, keeping blood clotting at bay. However, ADAMTS13 cannot act on VWF that is bound to platelet factor 4 (PF4). As such, it is of particular interest to note that a common feature between subjects presenting with VITT is high titres of antibodies against PF4. This raises the possibility that these antibodies preserve the stability of ultra-large VWF complexes, leading to the formation of endothelium-anchored VWF strings, which are capable of recruiting circulating platelets and causing uncontrolled thrombosis in terminal capillaries. Here, we share our viewpoint about the current understanding of the VITT pathogenesis involving the prevention of ADAMTS13’s activity on VWF by PF4 antibody-mediated stabilisation/ protection of the PF4-VWF complex.

## 1. Introduction

On March 2021, the European Medicines Agency announced that an extremely rare side effect of the ChAdOx1 nCoV-19 vaccine (AstraZeneca) is thrombosis accompanied by thrombocytopenia. The development of this vaccine was based on the use of a replication-deficient recombinant adenovirus vector generated from chimpanzees [1]. In April 2021, the research groups of Greinacher et al. and Schultz et al. presented clinical case reports describing cerebral venous sinus thrombosis with thrombocytopenia, as an extremely rare but serious side effect of this vaccine. The symptoms and manifestation of this phenomenon, termed vaccine-induced thrombotic thrombocytopenia (VITT), appears to resemble a condition known as autoimmune heparin-induced thrombocytopenia (HIT) with regards to clinical, laboratory and immunological features [2,3,4]. Besides the clinical manifestation of VITT approximately 5–22 days *post-vaccination*, auto-antibodies against Platelet Factor 4 (PF4) have been detected. However, the VITT patients (mainly in their 30–50’s) did not at any point receive heparin, which could potentially explain the symptoms. The cause and the origins of these PF4 auto-antibodies are presently unknown; however, it seems possible that these antibodies mimic heparin by binding to PF4, allowing the clustering of PF4 leading to the FcγRIIa-dependent platelet activation. Herein, we are proposing that autoantibodies against platelet factor 4 are a kind of “blocking antibodies” and are forming complexes with ultra-large von Willebrand factor multimers that are hyper-adhesive to platelets, and in consequence, platelets aggregate. This makes it impossible for ADAMTS13 to bind to VWF to initiate the proteolytic cleavage of VWF strings, in other words, which is then unable to regulate the multimeric size of the VWF. Furthermore, we will present how the autoantibodies against PF4 could mimic heparin by binding to PF4 and forming clusters with platelets. As a consequence, these complexes with platelet factor PF4 are promoting the persistence of endothelium-anchored ultra-large VWF strings which are capable of recruiting circulating platelets and causing uncontrolled thrombosis in terminal capillaries, by promoting aggregation, platelet consumption, and micro-thrombi formation within the capillary network (e.g., cerebral and/or splanchnic vein thromboses). Interestingly, Johnston et al. showed already in their report that PF4 was able to bind at multiple sites on VWF strings, and that the in vitro supplementation with ADAMTS13 was insufficient to prevent the forming of the earlier mentioned complexes [5]. This notwithstanding, we present in this commentary piece, a new point of view on VITT that connects ADAMTS13, PF4, and VWF.

## 2. ADAMTS13 and the Platelet Factor 4

**Figure 1:**
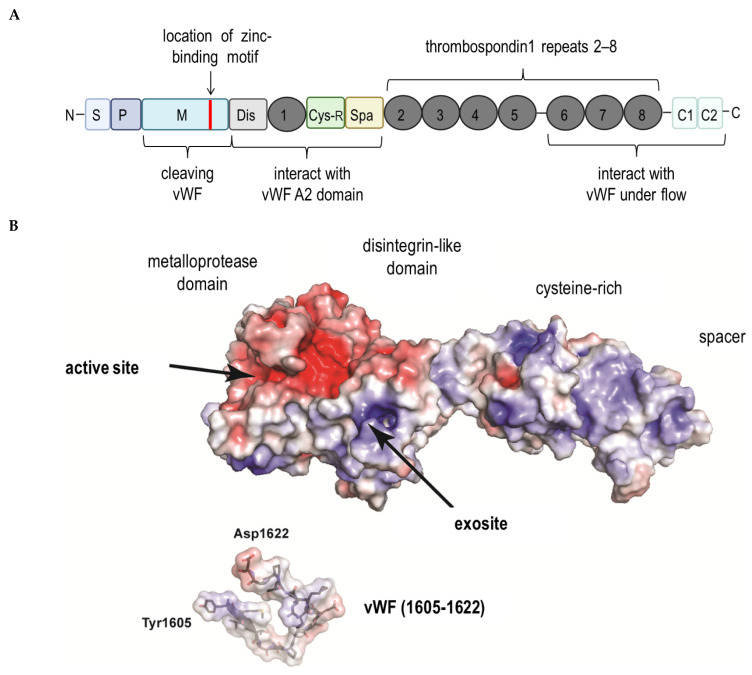
Representation of the ADAMTS13 structure. (**A**): The structural domains indicated are: signal peptide (S), propeptide (P), metalloprotease (M) (location of zinc-binding motif shown in red), disintegrin domain (Dis), first thrombospondin type 1 (TSP1) repeat (1), cysteine-rich domain (Cys-R), spacer domain (Spa), the second to eighth TSP1 repeats (2) through (8) and two CUB domains (C1 and C2). The metalloprotease domain is the catalytic center that cleaves VWF. The proximal carboxyl-terminal domains from Dis to Spa interact with the A2 domain of VWF. More distal carboxyl-terminal domains (TSP1 2–8) interact with VWF under fluid shear stress. (**B**): Structure of ADAMTS13 metalloprotease to spacer domains with a VWF peptide encompassing the scissile bond. The electrostatic surface is shown with positive charges in blue and negative charges in red.

**Figure 2:**
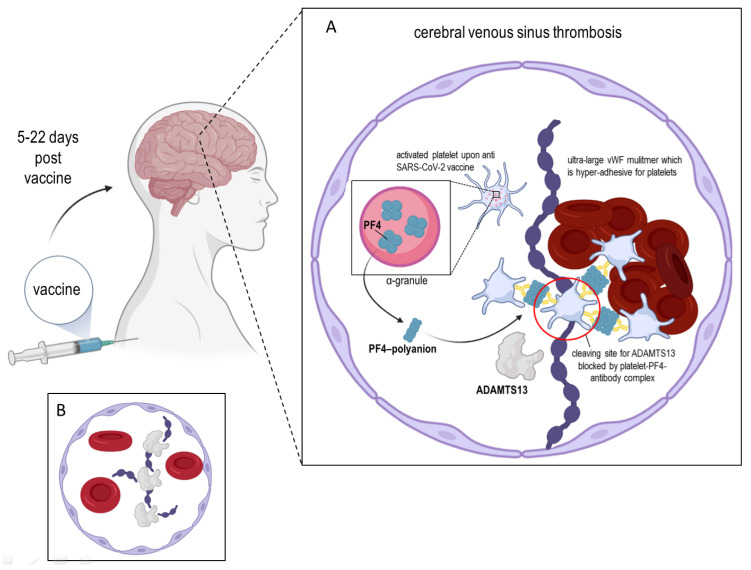
Scheme showing the possible link between ADAMTS13 and the vaccine-induced immune thrombotic thrombocytopenia. (**A**): In some rare cases, about 5–22 days post-vaccination against SARS-CoV-2 (ChAdOx1 nCoV-19), acquired autoantibodies against PF4 are forming complexes with ultra-large VWF multimers that are hyper-adhesive to platelets and lead to an inhibition of ADAMTS13 activity, which is then unable to regulate the multimeric size of the VWF. Here, we presented how the autoantibodies against PF4 could mimic heparin by binding to PF4 and forming clusters (red circle). In consequence, these complexes with platelet factor PF4 are promoting the persistence of endothelium-anchored ultra-large VWF strings which are capable of recruiting circulating platelets and causing uncontrolled thrombosis in terminal capillaries, by promoting aggregation, platelet consumption, and micro-thrombi formation within the capillary network. Thrombocytopenia appears to result from pronounced apoptosis and clearance of antibody-coated platelets additionally to consumption during the coagulation process. (**B**): Physiological functioning of ADAMTS13 which cleaves VWF strings to smaller, less adhesive multimers.

ADAMTS13, also known as VWF-cleaving protease, was isolated from blood plasma in 2001 by several independent research groups that also determined its partial amino acid sequence [6,7]. ADAMTS13 gene is located on chromosome 9q34 and contains 29 exons spanning 37 kb. The primary translation product consists of 1427 amino acid residues, comprising a signal peptide and a short propeptide, followed by a reprolysin-like metalloprotease domain, disintegrin domain, and first thrombospondin-1 (TSP1) repeat, and Cystein-rich and spacer domains. The more distal C terminus contains seven additional TSP1 repeats and two CUB (for complement C1r/C1s, Uegf, Bmp1) domains (Figure 1). Recently, Nazy et al. [8] reported that PF4 inhibits ADAMTS13 activity. They showed that PF4 suppresses ADAMTS13 activity when it binds to the VWF-A2 domain of the latter [8]. PF4, also known as Chemokine (C-X-C motif) ligand 4, is a small protein secreted primarily by activated platelets alpha granules (Figure 2A) [9] and also by monocytes [10] or microglial under neurodegenerating condition [11]. Mature human PF4 has a molecular weight of about 7.8 kDa and is a 70 amino acid protein that exists as a tetramer (Figure 2B) at physiologic pH and tonicity and has a high affinity for heparin [12]. Concentrations of PF4 after the activation of platelets is 0.4–2 μM in serum, a thousand-fold higher than in plasma, consistent with PF4’s platelet concentration [13]. As an important regulator of VWF proteolysis (via ADAMTS13), PF4 plays a crucial role in protecting VWF at the site of plug-formation and potentially augmenting thrombosis [8]. As described above, VITT patients exhibit symptoms which are comparable to a variant of autoimmune heparin-induced thrombocytopenia (aHIT), which is characterized by thrombocytopenia and thrombotic complications as an adverse reaction to heparin. Heparin-induced thrombocytopenia is a consequence of the action of pathogenic antibodies binding to PF4 and heparin complexes, leading to platelet activation and induction of a hypercoagulable state [14,15,16].

The main function of PF4 is to promote the coagulation of blood, but this cytokine also plays a role in innate and adaptive immunity when platelets are activated in response to infections. The promotion of coagulation is dependent on the affinity of PF4 for heparin and other glycosaminoglycans (GAGs) [17,18]. This occurs by PF4 neutralising the negatively charged heparan sulfate side chains of GAGs on the surface of platelets and endothelial cells. This facilitates platelet aggregation to form a thrombus. Consistent with its role, PF4 expression is raised after trauma to prevent blood loss from injury and also in response to infection [19,20]. Interestingly, PF4 plays a significant role in the clearance of viruses, CXCL4 KO mice exhibit reduced viral clearance from the lung compared to wild-type mice [21]. This feature is in agreement with the diminished innate immunity observed in the PF4 KO mice during early infections.

In addition, PF4 is involved in the pathology of a variety of inflammatory diseases including myelodysplastic syndromes [22], malaria [23], HIV-1 [24], atherosclerosis [25], inflammatory bowel disease [26], and rheumatoid [27] as well as psoriatic arthritis [28].

## 3. ADAMTS13 and the Von Willebrand Factor

The main role of ADAMTS13 is to cleave VWF, be they anchored on the surface of endothelial cells, in circulation, or at the sites of vascular injury. Von Willebrand factor is secreted by endothelial cells as highly prothrombotic ultra-large multimers. The shear-force of the blood stream stretches VWF multimers and unfolds its A2 domain [29]. The exposed Tyr1605-Met1606 bond in this domain lends itself to cleavage by ADAMTS13, consequently reducing the size of the VWF multimer and avoiding spontaneous platelet binding. Under normal conditions, ADAMTS13 maintains a closed conformation mediated by the interaction between the S domain and C-terminal CUB domains, and ADAMTS13 molecules are in the close surrounding of platelets or micro-aggregates of the later mentioned. In general, ADAMTS13 is able to cleave the VWF promptly; however, with regard to VITT, we propose that the formed complexes of PF4-antibodies, platelets and VWF are covering the cleavage site on the VWF strings, and in consequence, ADAMTS13 is unable to act adequately. However, upon binding to VWF via the ADAMTS13 CUB domains, a conformational change occurs on ADAMTS13 resulting in its transition from a closed to an open state [30,31,32]. A similar conformational change can also be instigated by the binding of anti-ADAMTS13 auto-antibodies at the CUB domains [30]. The interaction between these two proteins is mediated via numerous sites on both proteins and those at the A2 domain of VWF are particularly pertinent for the reduction of its multimeric size by the proteolytic activity of ADAMTS13. If these interactions are prevented for example by anti-ADAMTS13 inhibitory autoantibodies, the ultra-size VWF multimers would accumulate in the plasma, leading to the formation of platelet-rich microthrombi [32,33]. This could indeed be a possible contributor to the VITT disorder.

ADAMTS13 is synthesized primarily in the liver, and in human liver it is localized exclusively to hepatic stellate cells [34] which are considered to be the main source of ADAMTS13 in mammals. Additionally, vascular endothelial cells [35], megakaryocytes platelets [36], glomerular podocytes [37], tubular epithelial cells [37] as well as glial cells [38] are other potential sources of ADAMTS13. Platelets are specifically targeted to sites of vascular injury where they are activated and degranulated, releasing their contents including VWF. The components of granules exert prothrombotic and proinflammatory properties. However, concurrent local release of even small amounts of active ADAMTS13 protease can have profound inhibitory effects on thrombosis and inflammation [39].

The VWF is a large (∼270 kDa) hemostatic protein [40] produced in endothelial cells and megakaryocytes. Each monomer of VWF consists of identical repeated domains designated A to E [41,42]. The multimeric size of VWF is important for its function [43]. In the circulating blood, VWF is found as a series of multimers ranging from approximately 500 to 20,000 kDa, with the larger ones being hemostatically more effective. Hydrodynamic forces generated in the event of arteriolar bleeding triggers the activation of cleaved VWF, whereas uncleaved VWF is activated at lower physiological shear stresses and causes thrombosis. Histamine, thrombin, fibrin β-adregenic agonists, calcium ionophore A23187, desmopressin, and phorbol myristate acetate have all been demonstrated as stimulators of VWF secretion [44,45,46,47].

Von Willebrand factor mediates the binding of platelets to exposed collagen in areas of endothelial injury to promote normal hemostasis, which results in the formation of a platelet plug that stops bleeding [48]. It also participates in fibrin clotting by serving as a shuttle for the coagulation protein factor VIII (FVIII), by preventing its degradation by plasma proteases. Hence, the three major functional aspects of VWF are platelet binding, collagen binding, and FVIII binding. As soon as platelets are tethered to the injured endothelium by VWF, platelet activation and aggregation release platelet contents such as PF4, further contributing to clot formation [49,50]. The VWF string formation and elongation on endothelial surfaces are strongly regulated by ADAMTS13 through the proteolytic cleavage of VWF [51] or the inhibition of the formation of elongated VWF multimers by blocking the establishment of lateral disulfide bond [52,53]. The length and thickness of VWF multimers are strongly correlated with physiological hemostatic potential. Consequently, the cleavage or reduction of VWF multimers by ADAMTS13 plays an essential role in maintaining normal hemostasis. The lack of activity of ADAMTS13, in patients with mutations of ADAMTS13 or acquired auto-antibodies that block plasma ADAMTS13 activity, results in the formation of endothelium-anchored ultra-large VWF strings that are very effective in recruiting circulating platelets and causing uncontrolled thrombosis in terminal arterioles and capillaries.

The average concentration of VWF in blood plasma is around 10 μg/mL in humans [54]. Thrombin, histamine, fibrin, complement protein C5b-9 complexes as well as bacterial Shiga toxin can all induce the secretion of VWF from the Weibel–Palade bodies of endothelial cells [55,56]. Moreover, VWF release can be triggered at the sites of vascular injury or by desmopressin (DDAVP), which is used as a treatment for mild hemophilia A and von Willebrand disease [52,57], which is the most prevalent congenital bleeding disorder that arises from deficiencies in quantity or quality of von Willebrand factor (VWF). The quantitative deficiencies of VWF are considered to be either VWD type 1 (mild/moderate reduction of VWF) or type 3 (virtual absence of VWF). The estimated prevalence of VWD ranges from 0.1 to 1% of the population, but only approximately 1 in 10,000 individuals have clinically significant bleeding symptoms [58]. Pulmonary embolism and thrombocytopenia following ChAdOx1 vaccination. Of note, a case report about a 51 year old woman described severe symptoms of a pulmonary embolism and thrombocytopenia upon the ChAdOx1 vaccination, whereas the plasma coagulation factor activities among others those of VWF were in a physiological range [59]. All in all, it needs to be emphasized that VITT occurs in a very low incidence rate, and in general the risk of severe side effects of the currently available vaccines against SARS-CoV-2 can be estimated as low [60].

## 4. Conclusions and Final Remarks

In this commentary, we summarized the recent understanding about ADAMTS13 and provided our viewpoint on the potential impact of ADAMTS13 on VITT. We also provided explanation for the importance of this protein in the interplay between PF4 and VWF. There is no doubt that PF4 is a crucial contributor in the regulation of the proteolysis of VWF via ADAMTS13 and could play a role in the protection of VWF from proteolysis at the site of plug formation, too. It appears possible that in some susceptible patients PF4 become immunogenic either due to the shedding of heparan sulfate from damaged endothelial cells or perhaps to a component of the adenoviral vector-based vaccine. As consequence, the vaccine injection containing the adenovirus could cause micro-bleeding and the autoimmune effect. It is still elusive which component of the anti SARS-CoV-2 vaccine is involved in the process of forming antibodies against PF4. Interestingly, it has been reported that anti-PF4 antibodies which are causing VITT do not cross-react with the spike protein of SARS-CoV-2 [61]. Of note, the molecular mimicry should be taken into consideration when discussing the pathogenesis of PF4 autoantibodies, where the latter mentioned would bind to the FcRγIIA receptor of platelets, leading to their aggregation. Following this hypothesis, it appears to be possible for some susceptible vaccinated individuals that any DNA-vectored vaccine would develop an autoimmunity. Therefore, it would be worth investigating whether an adenoviral vector used for the development of vaccines triggers the onset of VITT. With regards to the very low prevalence of VITT, it would be important to know what the predisposing factors in this rare pathology are. Analysis of genetic and epigenetic links to VITT patients might uncover the cause that predisposes some individuals to the onset of VITT with these severe symptoms. Special attention ought to be paid to polymorphisms or mutations of ADAMTS13, particularly to the CUB domains, which would explain the inability of ADAMTS13 to cleave and reduce the multimeric size of VWF. To directly test the proffered hypothesis, the PF4 antibodies that are present in these subjects can be tested to ascertain whether they are indeed “blocking antibodies” that prevent the access of ADAMTS13 to VWF. It would also be important to ascertain the potential presence of autoantibodies to ADAMTS13 instead, in these subjects, as these could also prevent the cleavage of VWF multimers. Crystallographic determination of these potential contributors of VITT pathology, meaning PF4, VWF, and ADAMTS13 will help to uncover the mechanistic cascade of thrombus formation upon anti-SARS-CoV-2 vaccination. The identification of the predisposition factors and the dynamics of their interaction will provide new therapeutic strategies to respond effectively to VITT patients.
